# A Method for Evaluating Three-Dimensional Morphological Features: A Case Study Using *Marchantia polymorpha*

**DOI:** 10.3389/fpls.2019.01214

**Published:** 2019-10-02

**Authors:** Tomoyuki Furuya, Yoshitaka Kimori, Hirokazu Tsukaya

**Affiliations:** ^1^Department of Biological Sciences, Graduate School of Science, The University of Tokyo, Tokyo, Japan; ^2^Department of Imaging Science, Center for Novel Science Initiatives, National Institutes of Natural Sciences, Okazaki, Japan; ^3^Department of Management and Information Sciences, Faculty of Environmental and Information Sciences, Fukui University of Technology, Fukui, Japan; ^4^ExCELLS, National Institutes of Natural Sciences, Okazaki, Japan

**Keywords:** liverworts, *Marchantia polymorpha*, three-dimensional imaging, micro-computed tomography, mathematical image processing

## Abstract

The description and evaluation of morphological features are essential to many biological studies. Bioimaging and quantification methods have been developed to analyze the morphological features of plants. However, efficient three-dimensional (3D) imaging and its quantification are still under development, particularly for studies of plant morphology, due to complex organ structure with great flexibility among individuals with the same genotype. In this study, we propose a new approach that combines a 3D imaging technique using micro-computed tomography and a mathematical image-processing method to describe 3D morphological features. As an example, we applied this method to *Marchantia polymorpha*, a new model plant used for the evolutional study of land plants, and we evaluated a mutant individual with an abnormal 3D shape. Using this new method, we quantitatively described the thallus morphology of *M. polymorpha* and distinguished the wild type from a mutant with different morphological features. Our newly established method can be applied to various tissues or bodies with irregular 3D morphology.

## Introduction

The quantitative description of morphological features is essential for understanding the mechanisms of organogenesis. Recently, the development of bioimaging techniques and computational processing methods has allowed detailed and extensive imaging analyses in the field of plant morphology research, such that mathematical techniques have been adapted for the extraction of morphological features. For example, mathematical methods have been used to quantify the features of filament structures such as actin and microtubules (Boudaoud et al., 2014; Kimori et al., 2016; Furuya et al., 2018). Generally, in comparative analyses at the organ/tissue level, leaf morphology has been characterized using two-dimensional (2D) imaging, because leaves have flattened laminar shapes that allow large-scale analyses of leaf shape (Li et al., 2018). However, in reality, land plants have evolved three-dimensional (3D) bodies and 3D tissues (Graham et al., 2000; Ishizaki, 2017). Unlike animal body plans, in which organogenesis and pattern formation occur in a stereotypical manner, plant organogenesis is relatively flexible and can differ even among individuals with the same genotype. For example, the positioning of leaves within a tree differs significantly among individuals, making it difficult to quantify the 3D pattern in plants. Therefore, efficient 3D imaging and its quantification are needed for in-depth plant morphology studies.

Liverworts are basal land-plant lineage bryophytes that are grouped together with mosses and hornwarts (Wickett et al., 2014). Among liverworts, *Marchantia polymorpha* has attracted wide attention as a new model for the evolutional study of land plants due to the amount of available genetic information and efficient transgenic techniques (Ishizaki et al., 2016; Shimamura, 2016; Bowman et al., 2017). *M. polymorpha* forms a thallus as the main vegetative plant body during haploid gametophytic generation, which is the dominant form of generation in bryophytes. The thallus grows radially with repeated dichotomous branching via cell proliferation at the apical notch (Shimamura, 2016; Solly et al., 2017). Asexual reproduction is achieved via the gemma, a propagule formed in gemma cups on the thallus. The gemma is shaped like a disc with apical notches at both ends and grows laterally to form a flat, well-ordered gemmaling and thallus. Thus, the morphology of the wild-type (WT) thallus is generally easy to discern under certain growth conditions. As transgenic techniques have improved, however, many *M. polymorpha* mutants with abnormal 3D shapes and irregular warping or twisting have been isolated. Because such warping or twisting differs among individuals, quantitative description of the morphological features of these mutants is difficult. In this study, we applied a new method to describe 3D thallus shapes, combining a 3D imaging technique using micro-computed tomography (micro-CT) and a mathematical image-processing method. Micro-CT is a more suitable method to capture the 3D morphology with value data in tissue/individual level than other methods such as confocal microscopy, scanning electron microscopy, and stereo photography. This combined method will be applicable for various plant tissues of irregular complex forms.

## Materials and Methods

### Plant Materials and Culture Conditions

The *M. polymorpha* male accession Tak-1 was used as the WT line (Ishizaki et al., 2008). The knockout mutant line Mp*an-1^ko^* and its complementation line, *p*Mp*AN:*Mp*AN-1/*Mp*an-1^ko^*, were constructed by Furuya et al. (2018). WT and transgenic lines were cultured on half-strength Gamborg’s B5 medium (Gamborg et al., 1968) containing 1% (w/v) agar under 50–60 µmol m^−2^ s^−1^ continuous white light at 22°C.

### Fixation

Thalli were fixed overnight in FAA (50% (v/v) ethyl alcohol, 2.5% (w/v) glacial acetic acid, and 2.5% (w/v) formalin) and degassed. Fixed samples were incubated in 70%, 90%, and 100% (v/v) ethanol for 10 min each. Samples were stored in 100% ethanol and then dried lightly on paper immediately prior to micro-CT imaging.

### Micro-CT Imaging

Micro-CT images were obtained using the R_mCT2 micro-CT system (Rigaku) at 90 kV (180 µA) for 4.5 min with a 13-mm field of view. Fixed plant samples were placed on a plastic eraser as a pedestal.

### Extraction of the Plant 3D Image

**Figure 1:**
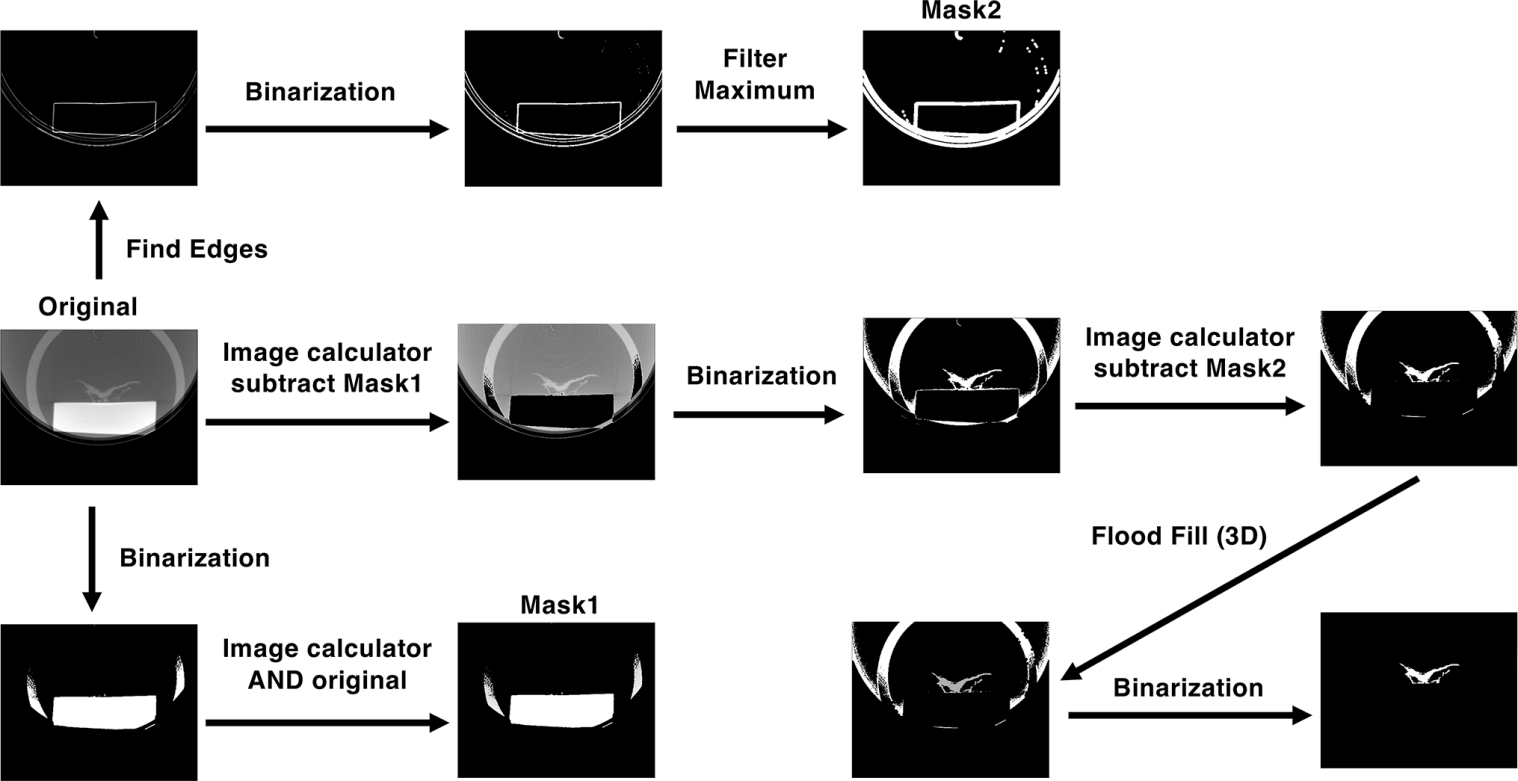
Processes of extracting the plant image from the micro-CT image. See Materials and Methods for details.

Micro-CT images were processed and reconstructed using ImageJ software (Schneider et al., 2012). A schematic diagram of the image-processing method is shown in **Figure 1**. Plant images were extracted from the micro-CT images using an image-processing program (**Supplementary Material**). We first extracted the part of the image with high signal intensity compared with that of the plant image, which contained the pedestal and part of a plastic case, by binarization. The extracted image was then combined with the original image using an image calculator to produce *Mask1*. To remove the pedestal image, the *Mask1* image was subtracted from the original micro-CT image. The obtained image was then binarized as shown in **Figure 1**. This binarized image retained the edge of the pedestal as noise. To remove this noise, we used another program to create *Mask2* by finding edges, binarizing, and filtering maxima; *Mask2* was then subtracted from the binarized image. In the subtracted image, the plant image was separated from other parts of the image. The plant image was then contrasted using a 3D flood fill algorithm. Finally, plant images were extracted by binarization.

### Calculation of Morphological Parameters

**Figure 2:**
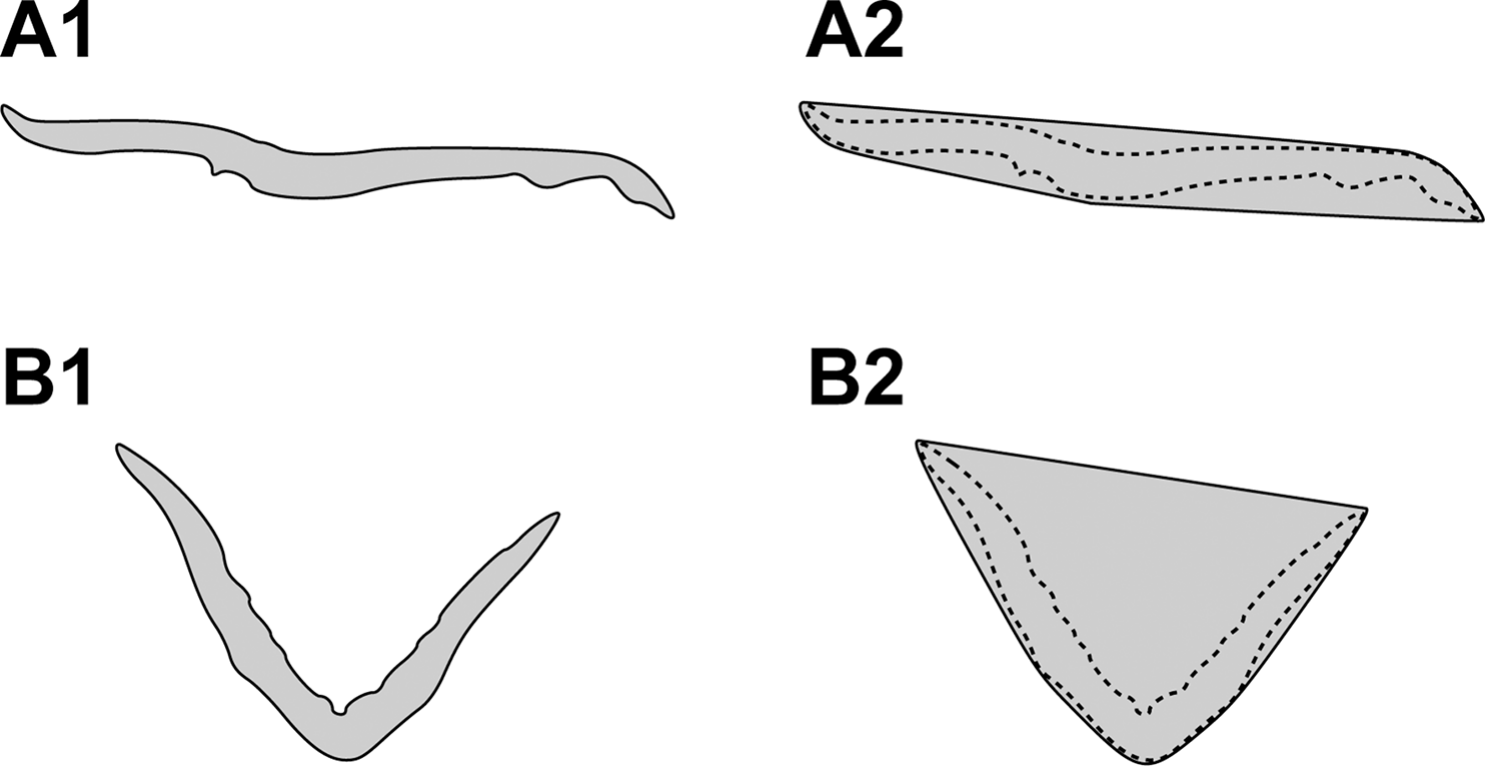
Cross-sectional two-dimensional (2D) slice extracted from the tomographic reconstruction of the plant body of Tak-1 **(A1)** and Mp*an-1^ko^* **(B1)**. Cross-sectional 2D slice extracted from the 3D convex hull of the plant body of Tak-1 **(A2)** and Mp*an-1^ko^* **(B2)**. The dotted lines denote the contours of each plant body.

To quantify the 3D morphology of the plant body, we utilized the following two morphological parameters in this study: convexity and solidity. These parameters are based on the computation of the convex hull of the plant body. **Figure 2A1 and B1** depict the cross-sectional 2D slice extracted from the tomographic reconstruction of the plant body of Tak-1 and Mp*an-1^ko^*, respectively. Each convex hull is shown in **Figure 2A2 and B2**.

The convexity *C* is defined as follows:

$$C=\frac{A_{C}}{A},$$

where *A*_C_ and *A* denote the convex and total surface areas of the plant body, respectively. Convexity is close to zero and one for non-convex 3D shapes and polygonal 3D shapes, respectively. When the 3D shape of the plant body deforms due to irregular warping, the actual surface area of the plant body becomes larger than that of its convex hull. Consequently, the convexity value decreases.

The solidity *S* is defined as follows:

$$S=\frac{V}{V_{C}},$$

where *V* and *V*_c_ denote the total and convex volumes of the plant body, respectively. As the whole plant body deforms due to irregular warping, the volume of its convex hull increases when compared with the volume of the actual plant body, whereas the solidity correspondingly decreases.

## Results

**Figure 3:**
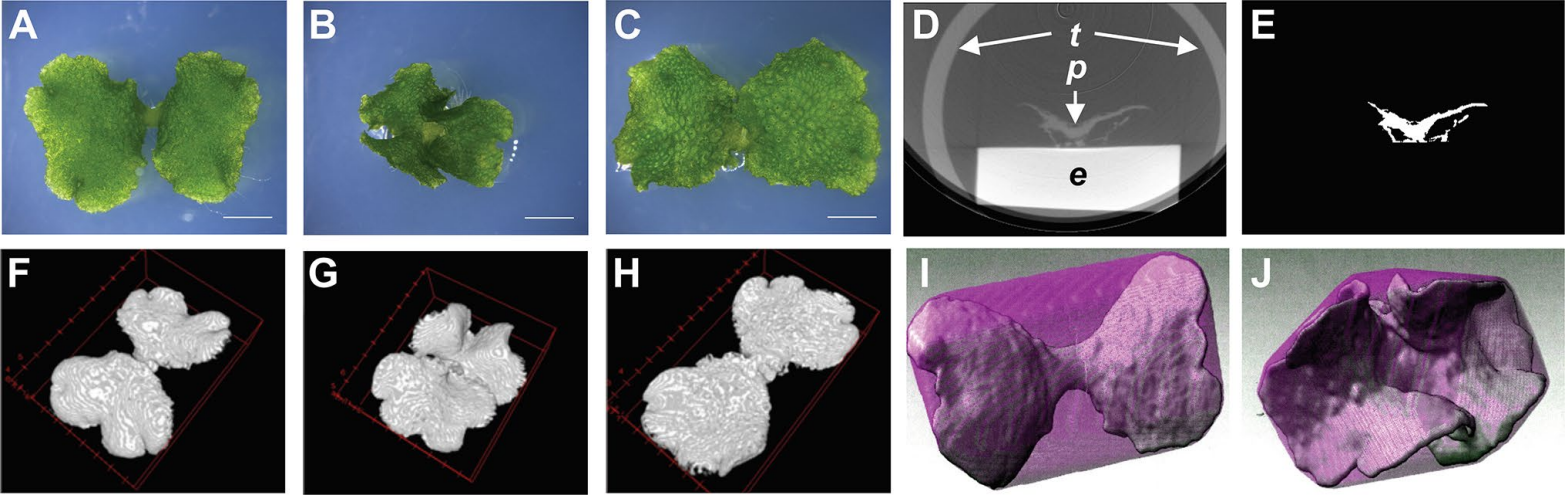
Cultured plant samples and its processing images. **(A–C)** Gemmalings (12 days old) of the wild-type line, Tak-1 **(A)**, the Mp*an* knockout line Mp*an-1^ko^* **(B)**, and the complementation line, *p*Mp*AN:*Mp*AN-1*/Mp*an-1^ko^* **(C)**. Bars, 3 mm. **(D, E)** Transverse views of a pre-processed micro-computed tomography (micro-CT) image **(D)** and the extracted plant image via manual image processing as shown in **Figure 1** **(E)**. *e* plastic eraser, *p* plant, *t* plastic tube. **(F–H)** Reconstructed three-dimensional (3D) images of Tak-1 **(F)**, Mp*an-1^ko^* **(G)**, and *p*Mp*AN:*Mp*AN-1/*Mp*an-1^ko^* **(H)**. **(I, J)** Convex hull image of reconstructed images of Tak-1 **(I)** and Mp*an-1^ko^* **(J)**. The convex hull image and extracted plant image are shown in magenta and green, respectively.

To establish a method to describe the morphological features of *Marchantia polymorpha*, we selected the Mp*an-1^ko^* morphological mutant for its characteristic thallus shape, which is twisted along the growth axis, in the 1- to 2-week-old gemmaling (Furuya et al., 2018). This phenotype is relatively uniform among individuals (**Figures 3A, B**). We also used the complementation line *p*Mp*AN:*Mp*AN-1/*Mp*an-1^ko^*, which has a morphology similar to that of the WT, Tak-1 (**Figure 3C**).

**Figure 4:**
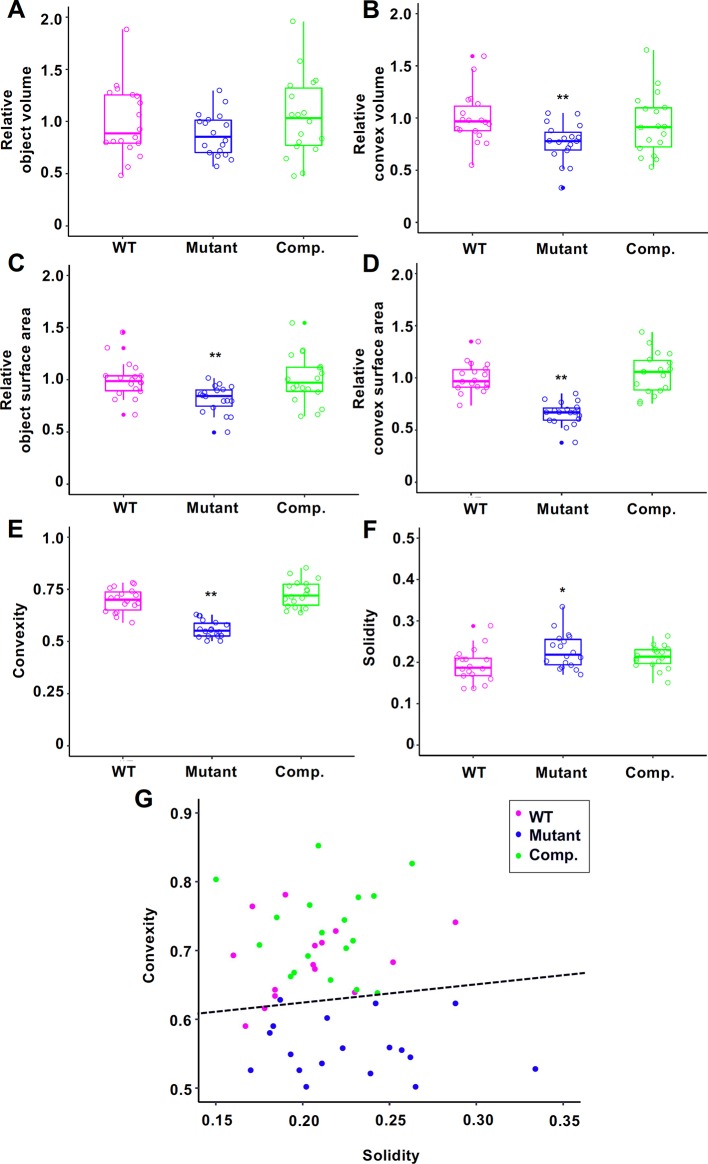
Morphological descriptors of 12-day-old gemmalings of Tak-1 (wild type (WT)), Mp*an-1^ko^* (Mutant), and *p*Mp*AN:*Mp*AN-1*/Mp*an-1^ko^* (Comp.) Relative object volume **(A)**, relative convex volume **(B)**, relative object surface area **(C)**, relative convex surface area **(D)**, convexity **(E)**, and solidity **(F)** are shown in box-and-whisker plots. Median values (middle bars) and first to third interquartile ranges (boxes); whiskers indicate the interquartile ranges of ∼1.5 Å; dots indicate outliers. The data in **(A–D)** are indicated as relative value to mean of Tak-1. Circles indicate value of each individual. Asterisks indicate significant differences compared with Tak-1 (*P < 0.05, **P < 0.01, Student’s *t*-test). *N* = 18. **(G)** Scatter plot of solidity versus convexity. Dashed black line is the classification boundary obtained by linear discriminant analysis using Tak-1 and Mp*an-1^ko^*.

For comparison, we captured 3D images of Tak-1, Mp*an-1^ko^*, and *p*Mp*AN:*Mp*AN-1/*Mp*an-1^ko^* using micro-CT. Based on previous observations (Furuya et al., 2018), we used 12-day-old gemmalings for micro-CT imaging. Plant samples were fixed using FAA solution and permuted using an ethanol series; micro-CT imaging was then conducted using a 100% ethanol-substituted specimen. Micro-CT images of plants were extracted from the captured images using ImageJ software (Schneider et al., 2012) (**Figures 1** and **3D, E**). These extracted images were reconstructed as 3D plant images (**Figures 3F–H**). To compare the morphological features among the lines, we used to calculate the convex hull polyhedron, which included all vertices of the 3D plant image as shape features (Neal and Russ, 2012). The convex hull of the extracted plant body was then calculated using the ImageJ 3D Convex Hull plugin (Sheets et al., 2011). Based on the 3D convex hulls of the plant bodies, shown as magenta surfaces surrounding plant bodies in **Figure 3I** (Tak-1) and **3J** (Mp*an-1^ko^*), we measured volumes and areas of objects and convex hull (**Figures 4A–D**). Then, we used these values to calculate two 3D shape descriptors: convexity and solidity (**Figures 4E, F**; Russ, 2011). Convexity defines the ratio of the convex surface area to the total surface area of the plant body, and solidity is the ratio of the whole volume to the convex volume of the plant body. The values of these descriptors are shown as scatter plot of solidity versus convexity in **Figure 4G**.

Mp*an-1^ko^* tended to have lower convexity values and larger solidity values than does the WT. To assess the validity of this method, we performed linear discriminant analysis (LDA) using the Tak-1 and Mp*an-1^ko^* results as training data (Duda et al., 2001). The discriminant function was given by *y* = 0.266*x* + 0.572, where *x* and *y* represent solidity and convexity, respectively. The accuracy rate for LDA was 0.917, and the kappa statistic was 0.834 (values range from 0 to 1). The complementation lines (*p*Mp*AN:*Mp*AN-1/*Mp*an-1^ko^*) were classified as WT based on the obtained discriminant function. These results indicate that the proposed method is a valid approach to discern plant groups with different morphological features.

## Discussion

In this study, we proposed a new method to describe the morphological features of the thallus of *M. polymorpha*. As the species name indicates, *M. polymorpha* exhibits wide variation in thallus morphology. In particular, mutations in genes responsible for morphogenesis often show very irregular thallus shape abnormalities; however, irregular twisting or warping is difficult to evaluate or analyze quantitatively. Our method is a simple and ideal approach for the analysis of irregular morphological defects. The development of our morphological assessment method consisted of three steps: sample preparation, extraction of 3D plant images from micro-CT data, and the selection of comparative factors.

In the preparation step, we first used fresh raw thalli to capture clear 3D micro-CT images. However, this process took at least 5 min per image, which seriously impacted our productivity. Because this method is not suitable for large numbers of samples, we fixed and stored the samples using FAA, followed by permutation in an ethanol series. These treated samples resulted in micro-CT images that were similar to those of fresh samples, with the advantage that they could be stored at room temperature.

In general, micro-CT images of a thallus sample also contain a pedestal, which introduces noise in the image analysis process (**Figures 1** and **3D**). In this study, we used a plastic eraser as the pedestal and a 15-ml plastic centrifuge tube as the case. When we used agar medium as the pedestal, the signal intensities were similar between plant and medium images; the reduced contrast between these images made it difficult to extract the sample image. Using the plastic eraser as a pedestal allowed us to obtain higher contrast between the sample and pedestal.

Comparing the 3D structures of the mutant and WT plant bodies, the mutant plant body was generally curved and exhibited a more undulating leaf surface than that of the WT. These morphological features were quantified by two 3D shape descriptors, convexity and solidity, which are defined mathematically in the *Materials and Methods*. Convexity represents the roughness of the plant body surface and is obtained by dividing the convex surface area of the plant body by its whole surface area. Thus, the measured convexity is low when the plant body is folded and the undulation of the surface increases. Solidity represents the concavity of the whole plant body and is calculated by dividing the volume of the plant body by its convex volume. As the plant body curls and the concavity of the whole plant increases, the convex volume increases, and consequently the solidity decreases.

Our method of describing morphology enables discernment of the 3D morphological features of *M. polymorpha*. This analysis is applicable to any 3D images, including the 3D distribution patterns of tree twigs and leaves, once the distribution has been scanned using 3D laser imaging. Although modifications to some steps (sample preparation, image processing, and comparative analysis) may be required depending on the sample type, our method can be applied to the 3D shapes of other plant tissues, especially in cases of irregular morphology.

## Data Availability Statement

All datasets generated for this study are included in the manuscript/**Supplementary Files**.

## Author Contributions

TF and YK performed the experiments. All authors designed the experiments, wrote the main manuscript text, and reviewed the manuscript.

## Funding

This work was supported by grants from the Ministry of Education, Culture, Sports, Science and Technology, Japan (Scientific Research on Priority Areas and Scientific Research on Innovative Areas) (no. 25113001 and 19H05672 to HT, no. 19K21189 to TF) and the NIBB Priority Collaborative Research Project (no. 17-526 to HT).

## Conflict of Interest

The authors declare that the research was conducted in the absence of any commercial or financial relationships that could be construed as a potential conflict of interest.
