# Supplementary material for: A Method for Evaluating Three-Dimensional Morphological Features: A Case Study Using Marchantia polymorpha
Source: Front Plant Sci. 2019 Oct 2;10:1214. doi: 10.3389/fpls.2019.01214 (PMC6783815; doi:10.3389/fpls.2019.01214)
Supplement: Supplementary file 1 [file Table_1.docx]

Supplementary Material

**An example for image processing of CT data**

**Process 1 (Macro #1)**

run("Image Sequence...", "open=/Users/XX/a_series_of_CT_images convert sort use");

# file > import > image sequence…, as an 8-bit file

selectWindow("a_series_of_CT_images");

saveAs("Tiff", "/Users/XX/Original.tif");

selectWindow("Original.tif");

close();

open("/Users/XX/Original.tif");

setAutoThreshold("Default dark");

//run("Threshold...");

setThreshold(135, 255); # please adjust according to image

//setThreshold(135, 255);

setOption("BlackBackground", true);

run("Convert to Mask", "method=Default background=Dark black");

saveAs("Tiff", "/Users/XX/Mask0.tif");

selectWindow("Mask0.tif");

close();

open("/Users/XX/Mask0.tif");

open("/Users/XX/Original.tif");

imageCalculator("AND create stack", "Original.tif","Mask0.tif");

selectWindow("Result of Original.tif");

saveAs("Tiff", "/Users/XX/Mask1.tif");

selectWindow("Original.tif");

run("Find Edges", "stack");

setAutoThreshold("Default dark");

//run("Threshold...");

setThreshold(135, 255); # please adjust according to image

//setThreshold(135, 255);

setOption("BlackBackground", true);

run("Convert to Mask", "method=Default background=Dark black");

run("Maximum...", "radius=2 stack");

saveAs("Tiff", "/Users/XX/Mask2.tif");

open("/Users/XX/Original.tif");

imageCalculator("Subtract create stack", "Original.tif","Mask1.tif");

selectWindow("Result of Original.tif");

setAutoThreshold("Default dark");

//run("Threshold...");

setThreshold(110, 255); # please adjust according to image

//setThreshold(110, 255);

setOption("BlackBackground", true);

run("Convert to Mask", "method=Default background=Dark black");

imageCalculator("Subtract create stack", "Result of Original.tif","Mask2.tif");

selectWindow("Result of Result of Original.tif");

saveAs("Tiff", "/Users/XX/Processed_image.tif");

**Process 2**

In the processed_image.tif, the plant image should be manually selected and colored using a 3D flood fill algorithm as shown in Figure 2.

Plugins > process > Foold Fill(3D)

**Process 3 (Macro #2)**

selectWindow("Processed_image.tif");

setAutoThreshold("Default dark");

//run("Threshold...");

setThreshold(60, 240); # please adjust according to image

//setThreshold(60, 240);

setOption("BlackBackground", true);

run("Convert to Mask", "method=Default background=Dark black");

saveAs("Tiff", "/Users/XX/Extracted_image.tif");

**Process 4**

Extracted_image.tif was used to calculate the morphological parameters using a plugin, 3D_Covex_Hull (Sheets et al., 2011).

Analyze > 3D Convex Hull > Measure all…
